# 4-Hydr­oxy-3-nitro­phenyl penta­noate

**DOI:** 10.1107/S1600536810002795

**Published:** 2010-01-30

**Authors:** Hongxiang Yang

**Affiliations:** a Tianjin Polytechnic University, Tianjin 300160, People’s Republic of China

## Abstract

In the title compound, C_11_H_13_NO_5_, an intra­molecular O—H⋯O hydrogen bond is formed between the hydr­oxy and the nitro groups, which results in the formation of a six-membered ring. The valer­oxy group shows a torsioned conformation, and connects to the aryl ring with a C—C—O—C torsion angle of 102.34 (1)°.

## Related literature

For general background to the use of phenolic esters as inter­mediates in organic synthesis, see: Trollsås *et al.* (1996 ▶); Svensson *et al.* (1998 ▶); Atkinson *et al.* (2005 ▶); Hu *et al.* (2001 ▶). For a related structure, see: Ji & Li (2006 ▶). For bond-length data, see: Allen *et al.* (1987 ▶).
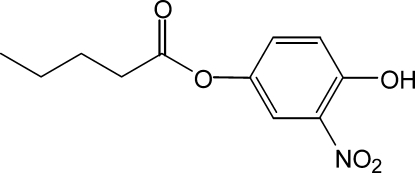


## Experimental

### 

#### Crystal data

C_11_H_13_NO_5_
*M* *_r_* = 239.22
Triclinic, 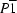

*a* = 5.3006 (14) Å
*b* = 10.435 (2) Å
*c* = 11.365 (3) Å
α = 67.340 (12)°
β = 81.074 (17)°
γ = 77.114 (16)°
*V* = 563.8 (2) Å^3^
*Z* = 2
Mo *K*α radiation
μ = 0.11 mm^−1^
*T* = 113 K
0.18 × 0.06 × 0.06 mm

#### Data collection

Rigaku Saturn CCD area-detector diffractometer
Absorption correction: multi-scan (*CrystalClear*; Rigaku/MSC, 2007 ▶) *T*_min_ = 0.980, *T*_max_ = 0.993
5175 measured reflections
2639 independent reflections
1972 reflections with *I* > 2σ(*I*)
*R*_int_ = 0.023

#### Refinement

*R*[*F* ^2^ > 2σ(*F* ^2^)] = 0.035
*wR*(*F* ^2^) = 0.100
*S* = 1.00
2639 reflections
158 parameters
H atoms treated by a mixture of independent and constrained refinement
Δρ_max_ = 0.24 e Å^−3^
Δρ_min_ = −0.27 e Å^−3^

### 

Data collection: *CrystalClear* (Rigaku/MSC, 2007 ▶); cell refinement: *CrystalClear*; data reduction: *CrystalClear*; program(s) used to solve structure: *SHELXS97* (Sheldrick, 2008 ▶); program(s) used to refine structure: *SHELXL97* (Sheldrick, 2008 ▶); molecular graphics: *SHELXTL* (Sheldrick, 2008 ▶); software used to prepare material for publication: *SHELXL97*.

## Supplementary Material

Crystal structure: contains datablocks global, I. DOI: 10.1107/S1600536810002795/bg2323sup1.cif

Structure factors: contains datablocks I. DOI: 10.1107/S1600536810002795/bg2323Isup2.hkl

Additional supplementary materials:  crystallographic information; 3D view; checkCIF report

## Figures and Tables

**Table 1:** Hydrogen-bond geometry (Å, °)

*D*—H⋯*A*	*D*—H	H⋯*A*	*D*⋯*A*	*D*—H⋯*A*
O3—H3⋯O2	0.872 (14)	1.871 (14)	2.6022 (13)	140.2 (13)

**Fig. 1.**
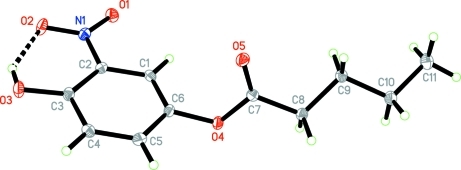
The molecular structure of the title molecule, with the atom-numbering scheme. Displacement ellipsoids are drawn at the 30% probability level. Hydrogen bond is shown as a dashed line.

## supplementary crystallographic information

### Comment

In organic synthesis, phenolic esters are useful intermediates (Trollsås *et al.*, 1996; Svensson *et al.*, 1998; Atkinson *et al.*, 2005; Hu *et al.*, 2001). We have synthesized the title compound according to the method in reference (Ji *et al.*, 2006), and we report herein its crystal structure.

In the molecule of the title compound (Fig. 1) bond lengths (Allen *et al.*, 1987) and angles are within normal ranges. An intramolecular O—H···O is formed between the hydroxy and the nitro groups, which results in the formation of a planar six-membered ring, coplanar with the aryl ring. The valeroxy group shows a torsioned conformation, the dihedral angle between aromatic ring and C6–O4–C7 being 99.64°. In addition valeroxy group is connected to the aromatic ring with a torsion angle (C5–C6–O4–C7) of 102.34 (1)°. A short O2···O2[1-x,1-y,2-z] = 2.855 (1)Å contact appears in the packing.

### Experimental

For the preparation of the title compound, 2-nitrohydroquinone pentanoate (325 mg, 1.0 mmol) was dissolved in chloroform (20 ml). At 273–278 K, anhydrous AlCl_3_ (200.2 mg, 1.5 mmol) was added to this solution, the reaction was stirred at room temperature for 1 h, and then hydrochloric acid (5 ml, 10%) was added. The reaction mixture was extracted with chloroform and dried with anhydrous sodium sulfate. After concentration, the residue was separated by flash column chromatography and purified by recrystallization from chloroform (yield; 167 mg, 70%, m.p. 310 K). Spectroscopic analysis: IR (KBr, ν, cm^-1^): 3267, 3096, 2960, 2937, 1762, 1539, 1238, 1138, 1099, 943, 840. Analysis required for C_11_H_13_NO_5_: C 55.23; H 5.48; N 5.85%. Found: C 55.32; H 5.54; N 5.79%.

### Refinement

C-H atoms were positioned geometrically, with C—H = 0.93 and 0.96 Å for aromatic and methyl H, respectively; and constrained to ride on their parent atoms. H3 coordinates were was further refined. In all cases *U*_iso_(H) = *xU*_eq_(C,*O*), where *x* = 1.2 for aromatic H and *x* = 1.5 for all other H atoms.

### Crystal data

**Table:**

C_11_H_13_NO_5_	*Z* = 2
*M**_r_* = 239.22	*F*(000) = 252
Triclinic, *P*1	*D*_x_ = 1.409 Mg m^−^^3^
Hall symbol: -P 1	Melting point: 310 K
*a* = 5.3006 (14) Å	Mo *K*α radiation, λ = 0.71073 Å
*b* = 10.435 (2) Å	Cell parameters from 1965 reflections
*c* = 11.365 (3) Å	θ = 2.1–27.9°
α = 67.340 (12)°	µ = 0.11 mm^−^^1^
β = 81.074 (17)°	*T* = 113 K
γ = 77.114 (16)°	Prism, colorless
*V* = 563.8 (2) Å^3^	0.18 × 0.06 × 0.06 mm

### Data collection

**Table:**

Rigaku Saturn CCD area-detector diffractometer	2639 independent reflections
Radiation source: fine-focus sealed tube	1972 reflections with *I* > 2σ(*I*)
graphite	*R*_int_ = 0.023
Detector resolution: 14.63 pixels mm^-1^	θ_max_ = 27.9°, θ_min_ = 2.2°
ω and φ scans	*h* = −6→6
Absorption correction: multi-scan (*CrystalClear*; Rigaku/MSC, 2007)	*k* = −13→13
*T*_min_ = 0.980, *T*_max_ = 0.993	*l* = −14→14
5175 measured reflections	

### Refinement

**Table:**

Refinement on *F*^2^	Primary atom site location: structure-invariant direct methods
Least-squares matrix: full	Secondary atom site location: difference Fourier map
*R*[*F*^2^ > 2σ(*F*^2^)] = 0.035	Hydrogen site location: inferred from neighbouring sites
*wR*(*F*^2^) = 0.100	H atoms treated by a mixture of independent and constrained refinement
*S* = 1.00	*w* = 1/[σ^2^(*F*_o_^2^) + (0.0553*P*)^2^] where *P* = (*F*_o_^2^ + 2*F*_c_^2^)/3
2639 reflections	(Δ/σ)_max_ = 0.001
158 parameters	Δρ_max_ = 0.24 e Å^−^^3^
0 restraints	Δρ_min_ = −0.27 e Å^−^^3^

### Special details

**Table:**

Geometry. All e.s.d.'s (except the e.s.d. in the dihedral angle between two l.s. planes) are estimated using the full covariance matrix. The cell e.s.d.'s are taken into account individually in the estimation of e.s.d.'s in distances, angles and torsion angles; correlations between e.s.d.'s in cell parameters are only used when they are defined by crystal symmetry. An approximate (isotropic) treatment of cell e.s.d.'s is used for estimating e.s.d.'s involving l.s. planes.
Refinement. Refinement of *F*^2^ against ALL reflections. The weighted *R*-factor *wR* and goodness of fit *S* are based on *F*^2^, conventional *R*-factors *R* are based on *F*, with *F* set to zero for negative *F*^2^. The threshold expression of *F*^2^ > σ(*F*^2^) is used only for calculating *R*-factors(gt) *etc*. and is not relevant to the choice of reflections for refinement. *R*-factors based on *F*^2^ are statistically about twice as large as those based on *F*, and *R*- factors based on ALL data will be even larger.

### Fractional atomic coordinates and isotropic or equivalent isotropic displacement parameters (Å^2^)

**Table:**

	*x*	*y*	*z*	*U*_iso_*/*U*_eq_	
O1	0.44132 (16)	0.16466 (9)	1.06926 (8)	0.0260 (2)	
O2	0.57276 (18)	0.35065 (8)	1.06023 (9)	0.0294 (2)	
O3	0.97659 (17)	0.43498 (8)	0.91208 (9)	0.0266 (2)	
H3	0.849 (3)	0.4414 (15)	0.9691 (14)	0.040*	
O4	1.05538 (15)	−0.03140 (8)	0.78112 (8)	0.0208 (2)	
O5	0.75270 (16)	0.06969 (8)	0.63771 (8)	0.0238 (2)	
N1	0.59085 (19)	0.24914 (10)	1.02548 (9)	0.0206 (2)	
C1	0.8163 (2)	0.11536 (11)	0.89438 (11)	0.0183 (3)	
H1	0.6925	0.0544	0.9283	0.022*	
C2	0.7982 (2)	0.23078 (11)	0.93060 (10)	0.0175 (3)	
C3	0.9768 (2)	0.32268 (11)	0.88102 (11)	0.0194 (3)	
C4	1.1752 (2)	0.29541 (12)	0.79260 (11)	0.0218 (3)	
H4	1.2982	0.3567	0.7566	0.026*	
C5	1.1953 (2)	0.18137 (12)	0.75690 (11)	0.0209 (3)	
H5	1.3315	0.1641	0.6969	0.025*	
C6	1.0158 (2)	0.09177 (11)	0.80900 (11)	0.0181 (3)	
C7	0.9099 (2)	−0.03100 (12)	0.69151 (10)	0.0178 (3)	
C8	0.9818 (2)	−0.16744 (12)	0.66970 (11)	0.0197 (3)	
H8A	1.0362	−0.2441	0.7506	0.024*	
H8B	1.1326	−0.1620	0.6056	0.024*	
C9	0.7641 (2)	−0.20491 (11)	0.62381 (11)	0.0210 (3)	
H9A	0.6992	−0.1255	0.5467	0.025*	
H9B	0.6189	−0.2196	0.6911	0.025*	
C10	0.8556 (2)	−0.33818 (12)	0.59220 (12)	0.0238 (3)	
H10A	0.9660	−0.3154	0.5108	0.029*	
H10B	0.9636	−0.4093	0.6601	0.029*	
C11	0.6337 (3)	−0.40135 (13)	0.58021 (14)	0.0332 (3)	
H11A	0.5280	−0.4282	0.6618	0.050*	
H11B	0.7039	−0.4851	0.5581	0.050*	
H11C	0.5260	−0.3316	0.5130	0.050*	

### Atomic displacement parameters (Å^2^)

**Table:**

	*U*^11^	*U*^22^	*U*^33^	*U*^12^	*U*^13^	*U*^23^
O1	0.0254 (5)	0.0286 (5)	0.0263 (5)	−0.0112 (4)	0.0048 (4)	−0.0114 (4)
O2	0.0387 (6)	0.0213 (4)	0.0310 (5)	−0.0050 (4)	0.0060 (4)	−0.0161 (4)
O3	0.0329 (5)	0.0241 (5)	0.0303 (5)	−0.0113 (4)	0.0036 (4)	−0.0170 (4)
O4	0.0237 (5)	0.0196 (4)	0.0229 (5)	0.0002 (3)	−0.0061 (3)	−0.0125 (3)
O5	0.0254 (5)	0.0229 (4)	0.0236 (5)	0.0022 (4)	−0.0063 (4)	−0.0108 (3)
N1	0.0229 (6)	0.0190 (5)	0.0191 (5)	−0.0016 (4)	−0.0013 (4)	−0.0076 (4)
C1	0.0187 (6)	0.0177 (5)	0.0192 (6)	−0.0047 (4)	−0.0034 (5)	−0.0058 (4)
C2	0.0185 (6)	0.0181 (5)	0.0154 (6)	−0.0012 (4)	−0.0015 (4)	−0.0066 (4)
C3	0.0239 (6)	0.0170 (5)	0.0187 (6)	−0.0027 (5)	−0.0049 (5)	−0.0074 (4)
C4	0.0214 (6)	0.0217 (6)	0.0224 (6)	−0.0069 (5)	−0.0014 (5)	−0.0067 (5)
C5	0.0201 (6)	0.0232 (6)	0.0181 (6)	−0.0013 (5)	−0.0011 (5)	−0.0077 (5)
C6	0.0221 (6)	0.0166 (5)	0.0174 (6)	0.0004 (4)	−0.0068 (5)	−0.0083 (4)
C7	0.0172 (6)	0.0234 (6)	0.0141 (6)	−0.0052 (5)	0.0017 (4)	−0.0085 (4)
C8	0.0206 (6)	0.0211 (6)	0.0199 (6)	−0.0015 (5)	−0.0023 (5)	−0.0111 (5)
C9	0.0208 (6)	0.0232 (6)	0.0216 (6)	−0.0042 (5)	−0.0015 (5)	−0.0111 (5)
C10	0.0269 (7)	0.0218 (6)	0.0258 (7)	−0.0041 (5)	−0.0040 (5)	−0.0115 (5)
C11	0.0348 (8)	0.0286 (7)	0.0441 (9)	−0.0095 (6)	−0.0049 (6)	−0.0189 (6)

### Geometric parameters (Å, °)

**Table:**

O1—N1	1.2264 (12)	C5—C6	1.3878 (16)
O2—N1	1.2461 (12)	C5—H5	0.9500
O3—C3	1.3476 (13)	C7—C8	1.4948 (15)
O3—H3	0.872 (14)	C8—C9	1.5171 (16)
O4—C7	1.3678 (15)	C8—H8A	0.9900
O4—C6	1.4032 (12)	C8—H8B	0.9900
O5—C7	1.2019 (14)	C9—C10	1.5258 (14)
N1—C2	1.4508 (14)	C9—H9A	0.9900
C1—C6	1.3675 (15)	C9—H9B	0.9900
C1—C2	1.3956 (15)	C10—C11	1.5170 (18)
C1—H1	0.9500	C10—H10A	0.9900
C2—C3	1.3987 (16)	C10—H10B	0.9900
C3—C4	1.3984 (16)	C11—H11A	0.9800
C4—C5	1.3762 (16)	C11—H11B	0.9800
C4—H4	0.9500	C11—H11C	0.9800
			
C3—O3—H3	109.7 (10)	O4—C7—C8	110.77 (10)
C7—O4—C6	117.46 (9)	C7—C8—C9	113.82 (9)
O1—N1—O2	122.30 (9)	C7—C8—H8A	108.8
O1—N1—C2	119.42 (9)	C9—C8—H8A	108.8
O2—N1—C2	118.27 (9)	C7—C8—H8B	108.8
C6—C1—C2	118.66 (10)	C9—C8—H8B	108.8
C6—C1—H1	120.7	H8A—C8—H8B	107.7
C2—C1—H1	120.7	C8—C9—C10	111.43 (9)
C1—C2—C3	121.70 (9)	C8—C9—H9A	109.3
C1—C2—N1	117.19 (10)	C10—C9—H9A	109.3
C3—C2—N1	121.08 (10)	C8—C9—H9B	109.3
O3—C3—C4	116.78 (10)	C10—C9—H9B	109.3
O3—C3—C2	125.67 (10)	H9A—C9—H9B	108.0
C4—C3—C2	117.55 (10)	C11—C10—C9	113.11 (10)
C5—C4—C3	121.13 (11)	C11—C10—H10A	109.0
C5—C4—H4	119.4	C9—C10—H10A	109.0
C3—C4—H4	119.4	C11—C10—H10B	109.0
C4—C5—C6	119.68 (10)	C9—C10—H10B	109.0
C4—C5—H5	120.2	H10A—C10—H10B	107.8
C6—C5—H5	120.2	C10—C11—H11A	109.5
C1—C6—C5	121.27 (10)	C10—C11—H11B	109.5
C1—C6—O4	119.95 (10)	H11A—C11—H11B	109.5
C5—C6—O4	118.54 (9)	C10—C11—H11C	109.5
O5—C7—O4	122.35 (10)	H11A—C11—H11C	109.5
O5—C7—C8	126.85 (12)	H11B—C11—H11C	109.5
			
C6—C1—C2—C3	0.58 (18)	C2—C1—C6—C5	−1.16 (18)
C6—C1—C2—N1	−177.40 (10)	C2—C1—C6—O4	173.22 (10)
O1—N1—C2—C1	−0.25 (16)	C4—C5—C6—C1	0.80 (19)
O2—N1—C2—C1	179.25 (11)	C4—C5—C6—O4	−173.66 (11)
O1—N1—C2—C3	−178.24 (11)	C7—O4—C6—C1	83.13 (13)
O2—N1—C2—C3	1.26 (17)	C7—O4—C6—C5	−102.34 (13)
C1—C2—C3—O3	−178.86 (12)	C6—O4—C7—O5	−0.14 (15)
N1—C2—C3—O3	−0.96 (19)	C6—O4—C7—C8	177.99 (8)
C1—C2—C3—C4	0.34 (18)	O5—C7—C8—C9	−28.67 (16)
N1—C2—C3—C4	178.24 (11)	O4—C7—C8—C9	153.30 (9)
O3—C3—C4—C5	178.56 (11)	C7—C8—C9—C10	175.07 (9)
C2—C3—C4—C5	−0.72 (18)	C8—C9—C10—C11	164.72 (10)
C3—C4—C5—C6	0.17 (19)		

### Hydrogen-bond geometry (Å, °)

**Table:**

*D*—H···*A*	*D*—H	H···*A*	*D*···*A*	*D*—H···*A*
O3—H3···O2	0.872 (14)	1.871 (14)	2.6022 (13)	140.2 (13)
